# RepetDB: a unified resource for transposable element references

**DOI:** 10.1186/s13100-019-0150-y

**Published:** 2019-01-29

**Authors:** Joëlle Amselem, Guillaume Cornut, Nathalie Choisne, Michael Alaux, Françoise Alfama-Depauw, Véronique Jamilloux, Florian Maumus, Thomas Letellier, Isabelle Luyten, Cyril Pommier, Anne-Françoise Adam-Blondon, Hadi Quesneville

**Affiliations:** URGI, INRA, Université Paris-Saclay, 78026 Versailles, France

**Keywords:** RepetDB, Transposable element, Database

## Abstract

**Background:**

Thanks to their ability to move around and replicate within genomes, transposable elements (TEs) are perhaps the most important contributors to genome plasticity and evolution. Their detection and annotation are considered essential in any genome sequencing project. The number of fully sequenced genomes is rapidly increasing with improvements in high-throughput sequencing technologies. A fully automated de novo annotation process for TEs is therefore required to cope with the deluge of sequence data.

However, all automated procedures are error-prone, and an automated procedure for TE identification and classification would be no exception. It is therefore crucial to provide not only the TE reference sequences, but also evidence justifying their classification, at the scale of the whole genome. A few TE databases already exist, but none provides evidence to justify TE classification. Moreover, biological information about the sequences remains globally poor.

**Results:**

We present here the RepetDB database developed in the framework of GnpIS, a genetic and genomic information system. RepetDB is designed to store and retrieve detected, classified and annotated TEs in a standardized manner. RepetDB is an implementation with extensions of InterMine, an open-source data warehouse framework used here to store, search, browse, analyze and compare all the data recorded for each TE reference sequence. InterMine can display diverse information for each sequence and allows simple to very complex queries. Finally, TE data are displayed via a worldwide data discovery portal. RepetDB is accessible at urgi.versailles.inra.fr/repetdb.

**Conclusions:**

RepetDB is designed to be a TE knowledge base populated with full de novo TE annotations of complete (or near-complete) genome sequences. Indeed, the description and classification of TEs facilitates the exploration of specific TE families, superfamilies or orders across a large range of species. It also makes possible cross-species searches and comparisons of TE family content between genomes.

## Background

Transposable elements (TEs) are major players in the structure and evolution of eukaryote genomes. Thanks to their ability to move around and replicate within genomes, they are probably the most important contributors to genome plasticity [1]. Indeed, genome size is generally correlated with TE abundance: with up to 90% of the genome consisting of TE sequences in some species, such as wheat [2] and wheat powdery mildew fungus [3] [4]. The difference of the genome size of *Zea luxurians* and *Zea mays* is directly correlated to the abundance of TE in these 2 genomes [5]. The insertion of TEs close to genes can affect gene structure, expression and function, contributing to the genetic diversity underlying species adaptation [6]. Many studies have shown that TEs are generally silenced through epigenetic defense mechanisms, and that these elements play an important role in epigenetic genome regulation [7].

TE detection and annotation is, thus, now considered essential for any genome study. With the development of new high-throughput sequencing technologies, a large number of genomes have been sequenced, resulting in very large amounts of sequence data. Automated TE de novo detection software can provide up to thousands of TE reference sequences per genome, generally in the form of consensuses. TEs are then classified into two classes, with various subclasses, orders and superfamilies defined on the basis of mechanistic, enzymatic and sequence similarity criteria. The two classes of TEs are defined on the basis of their transposition mechanisms: with (Class I) or without (Class II) an RNA intermediate [8]. TE identification often makes use of popular pipelines, such as RepeatModeler [9] and TEdenovo pipeline from the REPET package [10], including tools for the identification of repeats, their grouping into TE families, and the generation of multiple alignments to build TE consensus sequences. TE classification is usually based on similarity to known TEs and protein domains and/or according sequence structure [11].

Several databases for describing TE reference sequences already exist. Repbase Update [12, 13] contains (i) complete consensus sequences for TE families (70% of the database) built with homemade pipelines based principally on Recon [14] and LTR-FINDER [15], and (ii) TE copy sequences (complete or incomplete) extracted from individual genomic loci. DFAM [16], another database for repeats DNA families is based on profiles HMM constructed from multiple sequence alignment. The entries of Dfam are Repbase-derived library (humans, mouse, zebrafish, fly and nematodes). The Gypsy database (GyDB) [17] stores LTR-retroelements, including those of the Gypsy/Copia superfamilies in particular, and *Retroviridae*-like elements including sequences from the *Caulimoviridae* (plants Endoviruses). The SINE database (SINEBase) is dedicated to SINE elements prediction [18]. The P-MITE database hosts Miniature inverted-repeat transposable elements (MITEs) from 41 plant genomes [19]. The TIGR Plant Repeat Database [20] is a set of resources for the identification of repeats, including TEs, rDNAs, and telomere-associated sequences, populated with repeat sequences from 12 plant genomes from GenBank. This initial dataset is extended on the basis of sequence similarities between these sequences and GenBank sequences. The Transposable Elements Platform (TREP) is a curated TE database (http://botserv2.uzh.ch/kelldata/trep-db/index.html) mostly for monocotyledons and fungi. The MIPS Repeats database (PGSB-REdat) and Catalog (PGSC-REcat) are parts of the Plant Genome and Systems Biology platform (PGSB) including PlantsDB [21]. They contain TEs retrieved from TREP, TIGR repeats, Repbase and detected de novo in the genome sequences stored in PlantsDB. All these databases are accessed through web browsers and/or quick or advanced search forms. The curation of TE reference sequences is generally poor and based on automatic procedures.

However, intrinsic automated TE classification is an error-prone process. There was, therefore, an urgent need for annotation with TE consensus sequences together with the evidence justifying TE classification. None of the existing TE databases can provide the evidence on which the proposed classification is based. We filled this gap by developing the RepetDB database in the framework of GnpIS, a genetic and genomic Information System [22]. RepetDB is an instance of InterMine, a public open-source data warehouse that has been specially customized and enhanced [23, 24] with JBrowse [25] for searching, browsing, analyzing and comparing all the data provided for each TE consensus sequence in the many genomes analyzed. We use the powerful capacities of InterMine to create user-friendly interfaces allowing researchers to search and query data in multiple manners. Data can be exported in many commonly used formats, including fasta, GFF, BED or other tab-delimited formats. In addition, InterMine provides APIs in various languages, including Perl, Java, Python, Ruby, and JavaScript, for accessing its features via web services. The full capabilities of these web services are described at http://iodocs.apps.intermine.org/.

## Implementation

### InterMine instantiation and customization

RepetDB is an InterMine-based data warehouse. It was built with InterMine 1.4.2, with the InterMine web application used as the basis for data integration and presentation. Various comprehensive categories of reports through user-friendly interfaces for queries and visualization were developed with the templates provided by InterMine. RepetDB data integration is based on the InterMine data integration process built with Apache Ant 1.8.4, running in the Oracle Java 1.7.0_79 SE Runtime Environment and building a PostgreSQL 9.4.15 database. The RepetDB web application is deployed on Apache Tomcat 7.0.59 running in the same Java environment as used for data integration.

In addition to traditional InterMine configurations, some more advanced customizations were developed in Java, JSP (Java server page) and JavaScript. All of the RepetDB programs are hosted on a CentOS 6.8 virtual machine.

**Fig. 1:**
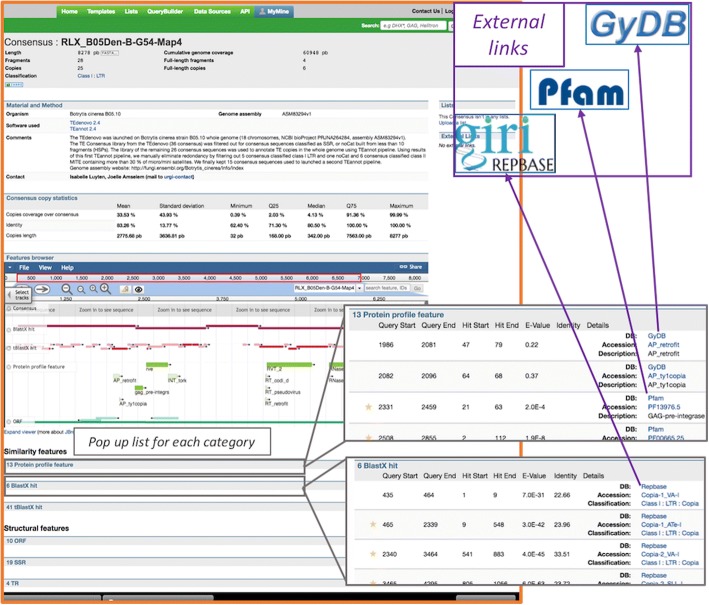
The RepetDB central object: the “Consensus card”, with magnifications of “protein profile” and “Blast hit” result categories with external links

The central object in RepetDB is the TE reference sequence, which is a consensus sequence. The main user interface visualization is a “TE reference page” referred to hereafter as the “Consensus card” (Fig. 1), on which information is gathered together and organized into five sections: (i) the “Header information” panel containing basic information, such as the consensus identifier, its length, a link to its sequence, its classification according to Wicker nomenclature [8] and basic occurrence metrics concerning annotation with this consensus in the genome (ii) a “Materials and Methods” section with a short description of the dataset (genome assembly version) and workflow used to provide the reference and other relevant information (from the point of view of the person submitting the dataset) (iii) a “TE copies statistics” section with various metrics concerning the genome copies annotated with the current TE reference, (iv) an embedded browser (JBrowse 1.12.3) displaying the sequence features on the consensus reference sequence and allowing the user to browse the tracks corresponding to all the structural and similarity-based results used by the TEdenovo pipeline to justify the TE classification proposed and (v) a detailed report corresponding to each track displayed in JBrowse. For each similarity to a protein profile or TE sequence, the coordinates of the alignment are displayed in a table. External links to the appropriate web page (i.e. PFAM, GYDB, Repbase) have been set up for the relevant identifier of hits based on similarity matching.

### An implementation suitable for standardized TE detection, classification and annotation

It is now common practice to propose gene structures with all the multiple sources of evidence used by the predictors to combine them. We have designed RepetDB to function in a similar manner. In databases such as Repbase, useful evidences such as coding sequence, terminal repeats evidences are usually presented in “full text description fields” of the consensus. In addition to this “description field”, DFAM, a database based on DNA profile prediction [16], also presents for each entry, the overlapping Repbase entries. The added value of RepetDB is that it displays the multiple sources of structural or similarity-based evidence used by PASTEC. Indeed, all the evidences used to automatically detect and classify TEs are presented by category in a text form, included coordinates and external links to Repbase, GyDB and PFAM and also displayed as annotation tracks in a Browser.

The TEdenovo pipeline [10] is used for the de novo detection of repeats. It uses Blaster [26], and groups repeats in a combined approach using Grouper [26], Recon [14] and Piler [27]. It then generates multiple alignments from the sequence clusters with MAP [28] and builds consensuses. Each consensus representing a structural variant of a TE family is classified with PASTEC [11], according to its structural and functional features (LTR, TIR, ORF, polyA tail), based on functional domain similarities to HMM profiles from the protein family databases PFAM [29] and GyDB [17]), and characterized TEs from the Repbase Update database [13]. Consensus sequences with no known structure or similarity are classified as noCat (i.e. “unknown”). Note that PASTEC may also be used on a TE library not provided by the TEdenovo pipeline.

RepetDB was also designed to integrate data relating to the annotation of TEs in a genome. The location of the TE copies (start, end) in the consensus and in the genome, are reported through the browser integrated into the RepetDB consensus card. A set of 26 optional metrics relating to the copies annotated are reported in a dedicated panel of the consensus card (e.g. cumulative coverage of the copies in the genome, number of copies, identity, copy lengths, copy coverage over the consensus (for the last three parameters: mean, standard deviation, median, 1st and 3rd quartiles, minimum, maximum)). The insertion of copy locations in RepetDB is based on standard GFF3 data formats (with the consensus used as the reference and the genome as the target) and a tabulated file (.tsv) containing the consensus copy statistics. When the TEannot pipeline is used, the PostAnalyse.py script (included in the REPET package) can be used to provide the statistics file in the appropriate format used by RepetDB (http://urgi.versailles.inra.fr/repetdb/docs/Data-submission.html#i.3.a.-consensus-copy-statistics). However, although any TE genome annotation pipeline can provide data in a suitable format for input into this system, the easiest way to insert data in RepetDB is to use outputs from the TEannot pipeline [30]. TEannot is based on three alignment methods (Blaster, Censor and RepeatMasker [31]). The HSPs obtained are filtered and combined. TE copies covered with SSRs over more than 75% (by default) of their length are then removed. Finally, a “long join procedure” [10] is used to address the problem of nested TEs. This procedure finds and connects fragments of TEs interrupted by other TEs inserted more recently, to build a TE copy. The nesting patterns of such insertions must respect three constraints: fragments must be collinear (both on the genome and the same TE consensus reference), of the same age (nucleotide identity with the consensus as a proxy) and separated by a more recent TE insertion.

### Data insertion and integration

The dataset metadata are integrated in RepetDB in xml format. TEdenovo and TEannot output files can be used for direct insertion, for each dataset: consensus sequences (fasta format), consensus classification file (tabulation-separated format), consensus annotation files including structural and functional annotations (GFF3 format). The data are displayed in the Consensus card JBrowse panel and textual feature categories.

Data integration occurs in two major steps: “integrate” and “post process”. The “integrate” step involves the loading of data from the provided files into the InterMine database. The “post process” step involves several operations, such as linking objects between data sources (e.g. linking consensuses to their wicker classification ontology terms), and data enrichment (e.g. fetching the NCBI taxonomy tree for all dataset organisms).

### TE query forms

**Fig. 2:**
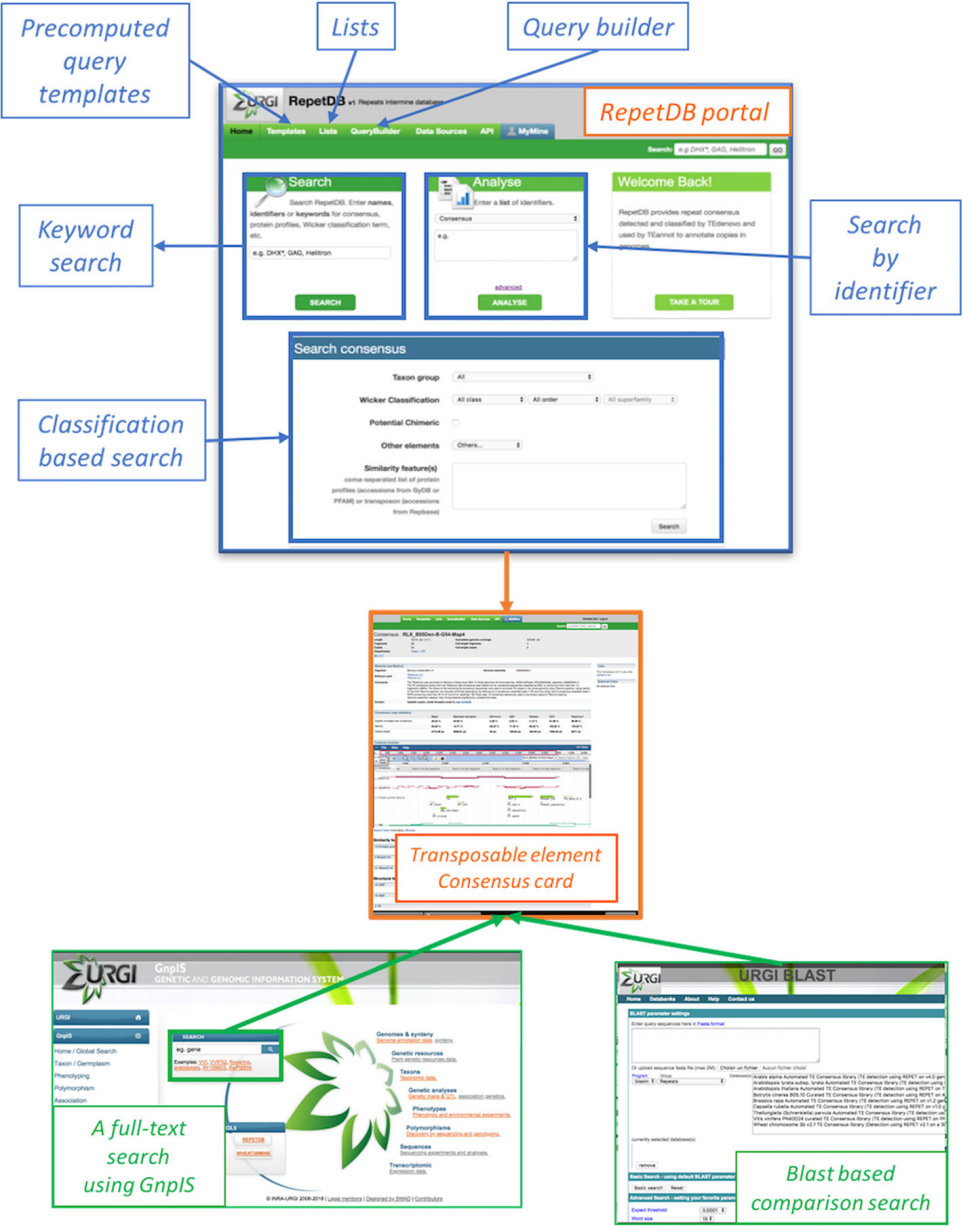
The different ways to query RepetDB data. Blue boxes are RepetDB internal forms and green boxes are external queries with results linked to RepetDB

We present various methods for querying RepetDB data (Fig. 2). Like any InterMine-based information system, RepetDB benefits from standard features, such as a keyword search on the main home page, a query builder, and template queries. One of the features of the InterMine-based system providing added value is the list management system, which makes it possible to create personal lists of data (e.g. a list of consensuses in response to a query) and to perform actions on these lists, such as unions, intersections and differences. In addition to the functions intrinsic to InterMine, the RepetDB home page contains a customized form enabling the user to search for repeats by organism (taxon group selection), by classification (Wicker classification [8]), potentially chimeric or other elements, such as virus-like elements) or by similarity features (e.g. protein profile features, blast hits on transposable elements databases, such as Repbase [13]). “Potentially chimeric” elements are defined as TEs with an ambiguous classification. We also present a full-text search with the GnpIS [22] data discovery tool (https://urgi.versailles.inra.fr/gnpis), which uses autocompletion to assist the user typing keywords for the search. The results are displayed in a table with the source of the data. Results can be restricted to one or several species. Finally, we also provide a web blast tool for searching for similarities to one or more consensus sequences present in RepetDB. All the TE consensus libraries are available through the URGI BLAST [32] server (https://urgi.versailles.inra.fr/blast/).

### Get data from RepetDB

RepetDB data export makes use of the default exporters of InterMine. Common standard formats, such as tabulated/csv-separated, fasta, BED, and GFF (https://genome.ucsc.edu/FAQ/FAQformat.html) are proposed on the query results and list analysis pages. The user can customize the table (addition or deletion of columns, column sorting etc.). For each data set, we provide a link to download the genome annotation performed with the consensus library of the dataset.

## Results

### TE reference sequences with annotation

TE reference sequences are available for 23 genomes in RepetDB, which currently stores 39,039 TE consensus. These TE consensuses were detected, classified and annotated in the framework of whole-genome sequencing projects or comparative genome analyses of transposable elements in various groups of species or organisms, such as the Brassicaceae family, Rosaceae, monocots and fungi (Table 1).

**Table 1:** Transposable element annotation metrics

Species	Genome assembly annotated (without gap)	Cumulative coverage	Genome coverage	No. of consensus sequences	No. of genome copies	No. of full-length genome copies
Brassicaceae						
*Arabidopsis lyrata*	206667935	76899516	37.21	2408	112563	9527
*Arabidopsis thaliana*	119146348	22954742	19.27	641	37129	2513
*Arabis alpina*	309171870	152175264	49.22	3204	268936	11729
*Brassica rapa*	283841084	101457103	35.74	2660	239373	10881
*Capsella rubella*	134834574	27975436	20.75	873	54560	3326
*Schrenkiella parvula*	123600562	19838473	16.05	455	37597	1356
Rosaceae						
*Fragaria vesca*	211673467	58062323	27.43	1543	112822	8576
*Malus domestica*	624851326	365363669	58.47	2456	564270	25280
*Prunus persica*	227411381	99590159	43.79	1738	170681	9056
*Pyrus communis*	577335413	194166715	33.63	975	482345	11435
Vitaceae						
*Vitis vinifera*	486205130	290981308	59.85	2473	475119	10551
Monocots						
*Triticum aestivum*	986092508	894245831	90.69	6671	785986	15905
*Zea mays*	2059701728	1768705851	85.87	7319	1381303	41666
Fungi						
*Blumeria graminis hordei*	87976437	59069666	67.14	733	122756	8909
*Botrytis cinerea B0510*	42630066	1583714	3.72	15	1927	263
*Botrytis cinerea T4*	37887365	254124	0.67	24	611	62
*Colletotrichum higginsianum*	50819261	3505545	6.90	41	1482	440
*Magnaporthe oryzae*	40949321	4549294	11.11	37	4358	463
*Melampsora larici populina*	97682699	49975736	51.16	1779	88708	6942
*Microtryum violaceum*	25201507	4423374	17.55	286	9620	640
*Puccinia graminis*	81521292	37620112	46.15	1625	69167	6648
*Sclerotinia sclerotiorum*	38001451	3459261	9.10	178	13868	622
*Tuber melanosporum*	123533734	73821108	59.76	905	72212	3845
Total				39039	5107393	190635

TE consensuses can be inserted into RepetDB via the TEdenovo [10, 11] and TEannot [30] pipelines, from the REPET package, which provides high-quality, standardized TE classification and genome annotation. The added value of TEdenovo stems from its ability to detect and build structural variants of TE families, representing their sometimes complex evolutionary dynamics [10]. In RepetDB, we store and provide access to the consensus built for each structural variant. The TE consensus library is used as an input of the TEannot pipeline, for the annotation of copies in the genome. For the automatic curation of some poorly defined consensuses, we run a second TEannot pipeline, using a library of consensuses filtered for sequences without full-length fragments or full-length copies in the genome. A copy may be built with one or more fragments joined by the TEannot long-join procedure. Depending on the context of TE annotation and the additional analyses performed, some of the datasets are manually curated and filtered for unreliable TE consensuses. The version, specific parameters and level of manual curation performed on the datasets are indicated in the “Comments” section of the datasets.

### Enhancing the discoverability of RepetDB data

This well-structured database should improve the visibility of the wealth of TE reference sequences already identified. In particular, its intrinsic functions, combined with its inclusion in the international data discovery network, make it possible to improve the FAIRness of these data [33]. Indeed, RepetDB ensures data accessibility, interoperability through the use of standard formats and ontologies, such as Wickers classification terminology, and reusability. Findability is increased through the GnpIS information system data discovery portal (https://urgi.versailles.inra.fr/gnpis), but the content of RepetDB is also indexed in various international discovery portals that are currently emerging in the field of plant biology. These portals can be used to search data with free keywords, across a set of databases displaying indices on several portals based on the same distributed full-text search technology and data model [34]. The content of RepetDB is currently available in the WheatIS data discovery tool for the international wheat research community (http://wheatis.org/Search.php) and from the IFB portal (https://urgi.versailles.inra.fr/ifb/), which aims to generalize the work of the wheat community to any plant.

## Discussion

### A database for the reuse of rich and often underexploited annotation data

The REPET package is one of the most widely used TE annotation tools in eukaryotic genome projects (mainly for plants and fungi). Its outputs are rich and often underexploited, not going beyond initial genome annotation in new genome annotation projects. The TE identification procedure provides access to large amounts of information for each reference sequence that are often lost after publication. We developed RepetDB for the storage and sharing of this information for other potential uses. The database is populated with the results of previous analyses already performed on species of agronomic interest in the framework of several unrelated projects. We group together results obtained in studies on plants (Brassicaceae, wheat, maize, grapevine, apple, oak, Rosaceae), symbiotic fungi and pathogens [35, 36]. However, there is no reason to limit the content of this system to the branches corresponding to these species. We will consider other eukaryotic species in the future. The system will be open to external submissions, making it possible to increase its diversity and the number of species represented.

### RepetDB to assist the pan-genome revolution initiated by third-generation sequencers

RepetDB is designed to be a TE knowledge base populated with full de novo TE annotations of complete (or near-complete) genome sequences. Continual improvements in sequencing technologies (which are now entering their third generation) over the last few years have made it possible to obtained genomic sequences at a cost, speed and quality close to those required for use as a routine procedure [37]. This should open up access to full-genome sequencing, which should no longer be restricted to a few representative individuals from a small number of species of interest.

Short-read resequencing, which generally provides no more than an inventory of SNPs and small indels, will probably be replaced by long-read sequencing to generate high-quality full-length genomic sequences opening up new possibilities for more detailed investigations of sequence polymorphism and diversity, to which TEs make an important contribution. We are entering the pan-genomic era, in which the search for dispensable genes in population-specific genomic sequences will be accompanied by the identification of new TE insertions. In this context, full gene and TE annotations of each individual genome will be required for subsequent analyses. The knowledge gained with one individual must be capitalized upon to guide the annotation of closely related genomes, and RepetDB is designed to make this possible.

## Conclusion

### RepetDB, a database of consistent TE datasets

RepetDB aims to provide a highly homogenized TE classification, with supporting evidence. We therefore integrated into the data submission process the systematic use of PASTEC [11], a tool from the REPET TEdenovo pipeline. This tool searches for evidence of TEs through dedicated similarity and structural searches, and uses this evidence to classify TEs automatically. We would therefore expect to obtain similar levels of sequence description and classification for different TEs in different species, thereby facilitating comparative genomics.

This consistency of description, classification and annotation makes it possible to perform consistent cross-species or cross-variety searches to compare TE family content between genomes. This tool can be used to explore specific TE families, superfamilies or orders across a large range of species.

Like any automatic procedure, full de novo TE annotation is likely to generate false positives, and ways of assessing correctness of TE classification relative to the corresponding reference sequence are therefore required. RepetDB is designed to display the evidence supporting each consensus classification, so as to facilitate the curation of TE reference libraries. Links to external databases are also available when similarities are detected to sequences from other databases (RepBase, GyDB [13, 17]).

## Availability and requirements

**Project name:** RepetDB

**Project home page:** http://urgi.versailles.inra.fr/repetdb/

The details of data submission are available from http://urgi.versailles.inra.fr/repetdb/docs/Data-submission.html

The RepetDB user guide is available from the RepetDB portal in the “Take a tour” panel or directly from http://urgi.versailles.inra.fr/repetdb/docs/User-guide.html.

**Operating system(s):** Any with a web browser

**Programming language**: Not applicable.

**Other requirements:** No.

**Any restrictions to use by non-academics:** No, RepetDB is publicly accessible
